# Suppressing molecular vibrations in organic semiconductors by inducing strain

**DOI:** 10.1038/ncomms11156

**Published:** 2016-04-04

**Authors:** Takayoshi Kubo, Roger Häusermann, Junto Tsurumi, Junshi Soeda, Yugo Okada, Yu Yamashita, Norihisa Akamatsu, Atsushi Shishido, Chikahiko Mitsui, Toshihiro Okamoto, Susumu Yanagisawa, Hiroyuki Matsui, Jun Takeya

**Affiliations:** 1 Department of Advanced Materials Science, Graduate School of Frontier Sciences, The University of Tokyo, 5-1-5 Kashiwanoha, Kashiwa, Chiba 277-8561, Japan; 2 Department of Applied Physics, Graduate School of Engineering, Osaka University, 1-1 Yamadaoka, Suita, Osaka 567-0047, Japan; 3 Chemical Resources Laboratory, Tokyo Institute of Technology, R1-12, 4259 Nagatsuta, Midori-ku, Yokohama, Kanagawa 226-8503, Japan; 4 PRESTO, Japan Science and Technology Agency (JST), 4-1-8 Honcho, Kawaguchi, Saitama 332-0012, Japan; 5 Department of Physics and Earth Sciences, Faculty of Science, University of the Ryukyus, 1 Senbaru, Nishihara, Okinawa 903-0213, Japan

## Abstract

Organic molecular semiconductors are solution processable, enabling the growth of large-area single-crystal semiconductors. Improving the performance of organic semiconductor devices by increasing the charge mobility is an ongoing quest, which calls for novel molecular and material design, and improved processing conditions. Here we show a method to increase the charge mobility in organic single-crystal field-effect transistors, by taking advantage of the inherent softness of organic semiconductors. We compress the crystal lattice uniaxially by bending the flexible devices, leading to an improved charge transport. The mobility increases from 9.7 to 16.5 cm^2^ V^−1^ s^−1^ by 70% under 3% strain. In-depth analysis indicates that compressing the crystal structure directly restricts the vibration of the molecules, thus suppresses dynamic disorder, a unique mechanism in organic semiconductors. Since strain can be easily induced during the fabrication process, we expect our method to be exploited to build high-performance organic devices.

## Abstract (web summary)

The mobility of organic semiconductors can be tuned by modifying their chemical composition or crystalline properties. Here, the authors show that bending organic single crystals increases their field effect transistor mobility due to restrained molecular vibrations and subsequently reduced dynamic disorder.

## Graphical abstract

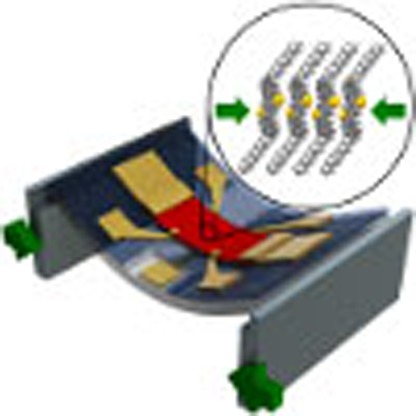

Organic semiconductors are already widely used in display applications and are expected to become even more ubiquitous when used as the building blocks for integrated circuits in applications such as radio-frequency identification tags or sensors^1^. An advantage over conventional semiconductors is their inherent softness and the possibility to be processed at low temperature, making them compatible with flexible plastic substrates. These properties stem from their weakly van der Waals (vdW) bonded molecular constituents. These bonds are also the origin of the large change of the crystal structure in response to a small mechanical force, for example, compressive strain. For inorganic semiconductors, the influence of strain on charge transport is very well-understood in terms of the modulation of the band structures and widely used in high-performance applications^2^. By contrast, the effect of strain on organic single-crystal semiconductors has rarely been investigated on a microscopic scale. At the same time, the field of organic semiconductors is shifting towards flexible applications. Therefore, understanding the relation between strain and device performance becomes indispensable.

Bending sensors using polycrystalline organic semiconductors have been reported for a range of material combinations^3^,^4^,^5^,^6^,^7^,^8^,^9^,^10^,^11^. Early studies found a mobility increase of about 10% on bending the substrates^3^,^4^,^5^,^6^,^7^,^8^,^9^. During bending, the polycrystalline layers are compressed in a non-uniform way, so that the grain boundaries, the weakest links in the semiconductor, are displaced the most, whereas the crystal structure itself is not altered. Indeed, later studies found that in polycrystalline layers, the effects are highly dependent on the grain size^10^,^11^. Therefore, polycrystalline thin films are not well-suited to study the physical origin of the modulation of transport properties as the exact amount of displacement is unknown. By contrast, single crystals, free of grain boundaries and other sources of static disorder^12^ have the great advantage that a macroscopic strain directly induces strain on the crystal lattice. Furthermore, single crystals are likely to exhibit intrinsic transport and have a high mobility compared with polycrystals. The effects of hydrostatic pressure and uniaxial strain on the mobility of vapour-grown rubrene single-crystal transistors have so far been investigated by several groups, and the change of mobility has basically been explained by the change of the overlap between adjacent molecular orbitals^13^,^14^,^15^,^16^. A similar conclusion was drawn from solution-processed thin films, where strain was induced during organic thin-film growth^17^,^18^. The physical vapour transport-grown single crystals use a non-scalable process and are therefore less relevant for applications. In recent years, the development of solution crystallized thin films^19^,^20^,^21^,^22^,^23^ has made it possible to build thin, homogeneous, high-purity and high-mobility organic single-crystal field-effect transistors. The homogeneity of these films provides a perfect platform to study the effect of uniaxial strain on the crystal structure and its influence on the molecular orbital overlap.

Here we show a large effect of strain on solution-processed single-crystal organic transistors on flexible substrates. Under homogeneous strain up to 3%, induced by bending the flexible substrates, the field-effect mobility increases significantly from 9.7 to 16.5 cm^2^ V^−1^ s^−1^. Contrary to expectations, the large increase of mobility cannot be explained by static compression of the crystal lattice. Therefore, we investigate the influence of molecular vibrations on charge transport. Density functional theory (DFT) calculations and temperature-dependent measurements support the hypothesis that the suppression of molecular fluctuations is a major mechanism behind the large mobility increase.

## Results and discussion

### Device properties without strain

**Figure 1:**
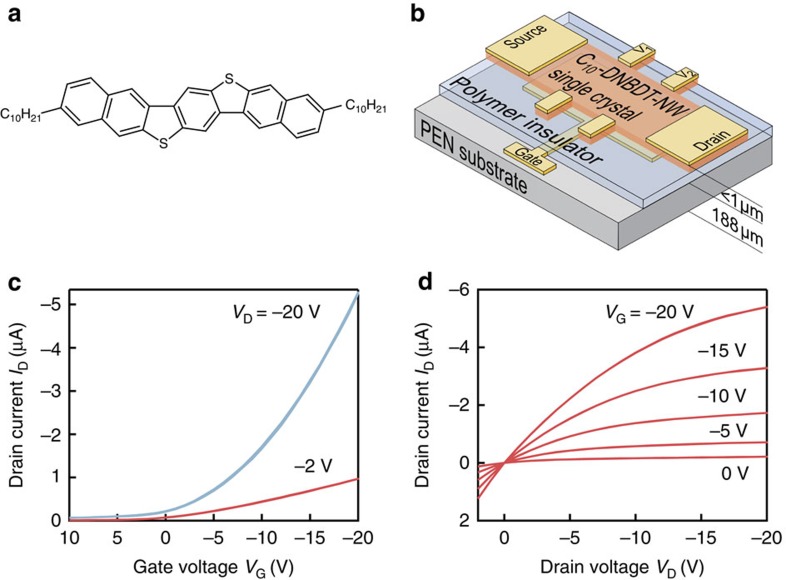
Experimental set-up. (**a**) Molecular structure of C_10_-DNBDT-NW. (**b**) Schematic of the bottom-gate top-contact organic field-effect transistor structure with the voltage probes for the four-terminal measurement. (**c**,**d**) Transfer and output curves for device A before applying strain.

**Figure 2:**
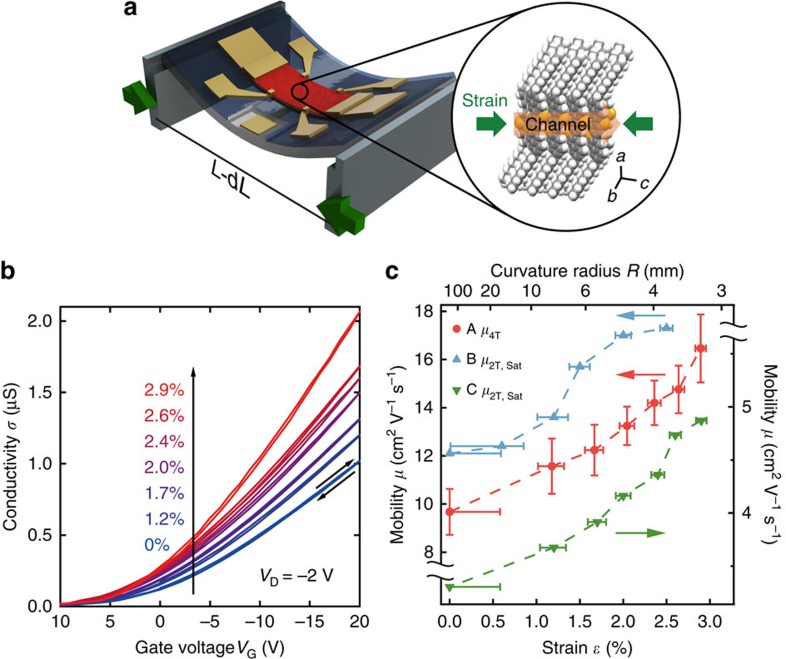
Evolution of charge transport while bending the substrate. (**a**) Schematics on how uniaxial strain was applied. (**b**) Evolution of the four-terminal conductivity under compressive strain for device A. The inclined arrows show the voltage sweep direction. (**c**) Evolution of the mobility under compressive strain. The average four-terminal mobility during the two compression cycles is shown for device A and the saturation mobility during the first compression for devices B and C. The horizontal error bars indicate the uncertainty of the applied strain deriving from the reading uncertainty of 0.1 mm of the compression distance d*L* when the bending set-up is used, and the vertical error bars for device A represent the s.d. of the mobility during the two compression cycles.

To investigate the effect of strain on charge transport in organic single-crystal semiconductors, a solution crystallized thin film of 3,11-didecyldinaphto[2,3-*d*:2′,3′-*d*′]benzo[1,2-*b*:4,5-*b*′]dithiophene (C_10_-DNBDT-NW) was used (Fig. 1a) to fabricate field-effect transistors (Fig. 1b)^24^. Single-crystal C_10_-DNBDT-NW exhibits a very high hole mobility of up to 16 cm^2^ V^−1^ s^−1^. Hall effect measurements indicate that holes in C_10_-DNBDT-NW are delocalized over many molecules^24^. Moreover, due to the alkyl side-chains, C_10_-DNBDT-NW is solution processable. To obtain large-area single-crystal thin films, we used the edge casting method^19^,^22^. The crystal was grown parallel to the channel, so that the *c*-axis is the transport axis (Fig. 2a)^24^. We confirmed that the channel consists of a single crystal devoid of grain boundaries using a polarized optical microscope. The transfer and output curves of device A before applying strain are shown in Fig. 1c,d. Neither the output nor the transfer curves show any injection limitations. Four-terminal measurements confirmed the contact resistance to be negligible. To induce strain, the organic field-effect transistor (OFET) was bent using a self-developed set-up (Fig. 2a, Supplementary Figs 1 and 2). When a flexible substrate is bent, its inner surface is compressed while its outer surface is stretched. Inside the substrate exists a neutral layer, which is neither stretched nor compressed. The surface strain *ɛ* was calculated using the following equations^25^,^26^:


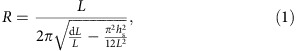



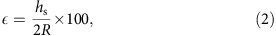


where *R* is the curvature radius, *L* is the substrate length, d*L* is the compression distance with *L*−d*L* being the horizontal distance from one side to the other after bending and *h*_s_ is the substrate thickness. The surface strain of the single-layer substrates was confirmed to follow these equations^27^. Since we employ a multilayer structure using materials with a wide range of elastic moduli, it is not *a priori* clear if the above equations are applicable. A more involved assessment of the position of the neutral layer^28^ shows a small shift of the neutral layer due to the additional gold and insulator of 0.5 μm at most, which is negligible compared with the overall thickness of the substrate (188 μm). This result shows that devices considered here can be approximated by a single-layer structure, whose thickness is given by the thickness of the substrate *h*_s_ (Fig. 1b). We also confirmed that the strain calculated by the equations is consistent with the strain measured by X-ray diffraction (XRD) as discussed later. We bent the substrate in such a way that the semiconductor film is compressed along the channel direction. Assuming that a single crystal of C_10_-DNBDT-NW has an elastic modulus similar to other organic semiconductors of ∼15 GPa^29^,^30^, the equivalent pressure induced was slightly <0.5 GPa with a strain of 3%, which is comparable to what is achieved in high-pressure cells^14^,^15^.

### Evolution of charge transport under applied strain

The modulation of charge transport was measured while increasing the curvature by bending the substrate. Several devices were measured with a maximum compressive strain close to 3%. This strain corresponds to a bending radius of the substrates of about 3 mm. Up to a strain of 2.5%, the semiconductor films show only marginal changes under an optical microscope, whereas above 2.5% large cracks appear and devices start to fail (Supplementary Figs 3 and 4). The four-terminal conductivity *σ* of device A shows a clear reproducible increase with negligible hysteresis (Fig. 2b). We compressed and decompressed the device twice and confirmed that the conductivity increases monotonically as a function of the compressive strain. The four-terminal mobility was extracted at *V*_G_=−20 V in the linear regime to exclude any effects from changes in the contact resistance. The average mobility during the two compression cycles increased monotonically under compressive strain (Fig. 2c). The four-terminal mobility increased by 70% from 9.7 to 16.5 cm^2^ V^−1^ s^−1^ under a maximum compressive strain of 2.9%. A hysteresis in the extracted mobility is visible between the compression and decompression step, but this is much smaller than the magnitude of the change in mobility (Supplementary Fig. 5), meaning that the mobility modulation is essentially reversible and reproducible. We note that the two-terminal mobility extracted in the linear regime increased by a factor of 1.6, and the one extracted in the saturation region increased by a factor of 1.8 (Supplementary Fig. 6). For comparison, the two-terminal saturation mobility of device B and device C is plotted as well in Fig. 2c. The mobility increases by about a factor of 1.4–1.5 under strain (Supplementary Fig. 7). Devices B and C show no injection limitation, suggesting the contact resistance is negligible as well. These results lead to the conclusion that in C_10_-DNBDT-NW indeed the mobility substantially increases under compressive strain.

**Figure 3:**
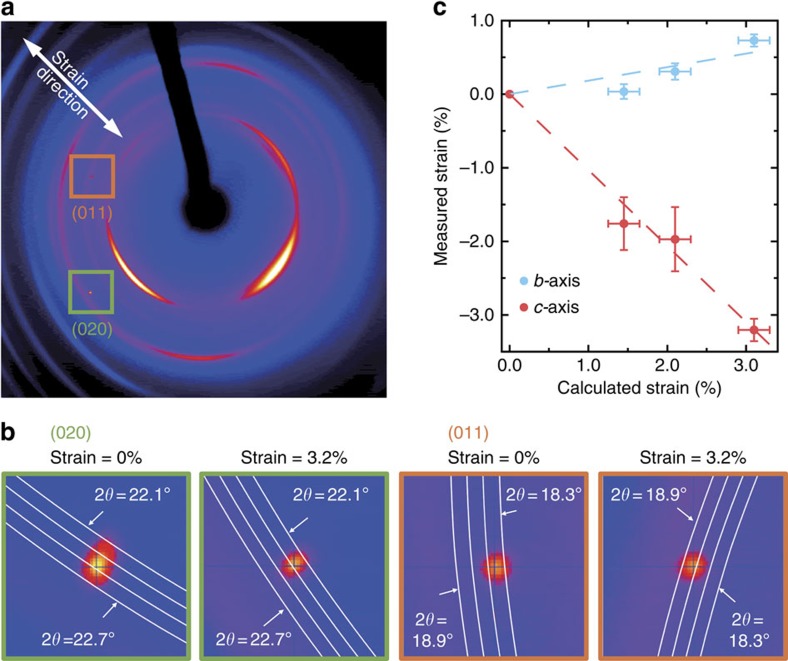
Crystal structure under strain. (**a**) Transmission XRD patterns of a strained single-crystal semiconductor thin film (calculated strain=3.2%). The broad ring-like features stem from the PEN substrate and the polymer insulator (Supplementary Fig. 8). Clearly distinct single peaks are observed, confirming that the thin films are in fact single crystals. (**b**) When strained, the 011 and 020 peaks shift from 22.467° to 22.282° and from 18.434° to 18.741°, respectively, confirming that the strain is applied homogeneously throughout the single crystal. (**c**) Decrease of the *c*-axis and elongation of the *b*-axis under strain. The bottom axis shows the applied strain along the *c*-axis calculated from the bending radius and equation (2). The left axis is the induced strain along the *b*- and *c*-axes measured by XRD. The horizontal error bars show the uncertainty of the calculated strain originating from the sample holder design. The vertical error bars are the s.d. of the measured strain stemming from the variation of the diffraction peaks among different crystal domains and multiple measurements.

To discuss the origin of the large mobility increase, we first need to exclude several extrinsic effects. First of all, the measured OFETs are not dominated by contact resistance, as the four-terminal mobility from device A shows the same increase as the two-terminal mobility. Furthermore, the capacitance of the gate insulator and therefore accumulated charge carriers in the channel were assumed to remain the same under strain. Due to the Poisson effect, the thickness of the gate dielectric is expected to slightly increase under compressive strain, meaning less charge carriers are accumulated in the channel. Therefore, the assumption of a constant capacitance means that the measured mobility increase is slightly underestimated. The channel length and width are assumed to be constant, since their geometrical modulation is similar to the extent of the strain, which is <3%. Therefore, we can exclude extrinsic factors like contact resistance and small changes in the thickness of the gate dielectric to be the origin of the large mobility modulation. As we are employing single-crystal thin films, grain boundaries can be excluded, too. Therefore, the strain is applied homogeneously over all molecules, which is confirmed by XRD measurements (Fig. 3a,b). Thus, the origin of this large increase has to be attributed to the changes in the crystal structure itself.

### Influence of strain on crystal lattice and band structure

To study the influence of changes in the crystal structure on charge transport, we first directly measured the change of the lattice constants under strain by XRD experiments. The measured strain along the *c*- and *b*-axes is plotted as function of the strain along the *c*-axis calculated from the bending radius and equation (2) in Fig. 3c. The consistency between the experiment and the calculation indicates that the simple equations give a good estimation of strain even for the multilayer devices in this study. At the same time, the XRD measurement revealed that the *b*-axis expands by 0.55% under 3.0% strain along the *c*-axis. Based on these lattice constants, we performed vdW-DFT calculations to determine the equilibrium crystal structure under ambient conditions and under strain using the Vienna Ab-initio Simulation Package (VASP)^31^,^32^,^33^. The calculated crystal structure under ambient conditions differs by <0.1% from the measured crystal structure reported before^24^, highlighting the accuracy of the chosen calculation method.

Under strain, the intermolecular distance is reduced by 3% along the *c*-axis and increased by 0.55% along the *b*-axis (Fig. 3c), leading to a slight rotation of the molecules. As mentioned above, charge carriers in single-crystal C_10_-DNBDT-NW are known to be delocalized over several molecules^24^. Therefore, it is reasonable to discuss the charge transport in terms of band-like transport. We calculated the band structure under ambient condition and under applied strain. The bandwidth of the HOMO band along the *c*-axis expands from 0.562 eV without strain to 0.624 eV under a 3% compressive strain. The effective mass, however, even slightly increases from 0.877 to 0.882 under 3% strain. The charge carrier mobility *μ* in the band transport model is described by *μ*=*eτ*/m*, where *e* is the elementary charge, *τ* is the relaxation time of the charge carriers and m* is the effective mass. Since the mobility is inversely proportional to the effective mass, we conclude that static changes of the strained crystal structure slightly reduce the mobility but overall only marginally influence charge transport properties. This means that the large mobility increase cannot be explained by the static changes of the crystal structure.

### Calculating molecular fluctuations

**Figure 4:**
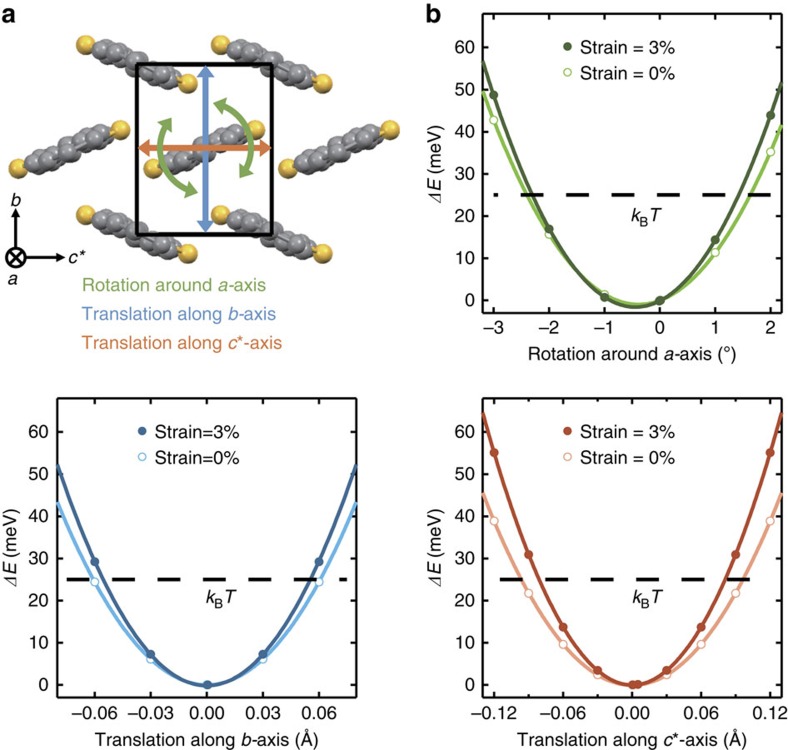
Suppression of dynamic disorder under uniaxial compressive strain. (**a**) DFT calculations were used to study the translational and rotational energy of a single molecule in the crystal lattice. (**b**) Under a 3% strain, the amplitude of the harmonic oscillation of the molecule is suppressed by about 8.7–16% (translation along *b* and c*) and 7.5% (rotation around *a*). This is equivalent to a temperature reduction by 50–85 K (translation along *b* and c*) and 45 K (rotation around *a*).

The mobility in the band transport picture is proportional to the relaxation time. Therefore, the large increase of the mobility might have its origin in a drastically reduced scattering of charge carriers. Organic molecules are weakly bonded by vdW interaction and therefore molecular fluctuations are much larger compared with inorganic semiconductors, leading to large temporal fluctuations of the transfer integral, which are in the same order of magnitude as the transfer integral itself^34^. This results in a smaller relaxation time of the charge carrier in organic semiconductors. Therefore, we infer that the mobility increase has to stem from the reduction of the thermal fluctuations, that is, the dynamic disorder. To prove this, a straightforward simulation was performed. The total energy of a system comprised of the core of seven neighbouring C_10_-DNBDT-NW molecules was calculated using the Gaussian Software package^35^. Then, the central molecule was translated or rotated by a certain amount and the total energy of the system was recalculated (Fig. 4a). These steps were carried out for the unstrained, as well as strained crystal structure. The results show a quadratic dependence of the energy on the displacement along the *b*- and c*-axes, as well as the rotation angle around the *a*-axis, leading to harmonic oscillations (Fig. 4b). The clear increase in potential energy in the strained crystal structure leads to a reduction of the oscillation amplitude of each molecule, essentially suppressing dynamic disorder. The amplitude of the oscillation was estimated by using the equipartition theorem where the average energy of each degree of freedom is given by <*E*>=*k*_B_*T*/2. Hence, the amplitude is reduced by 8.7–16% for the translations and by 7.5% for the rotation. This reduction of the amplitude of the molecular vibration is equivalent to a reduction of temperature by 45 K for the rotation around the *a*-axis, 50 K for the translation along the *b*-axis and 85 K along the c*-axis.

### Temperature dependence of the mobility

**Figure 5:**
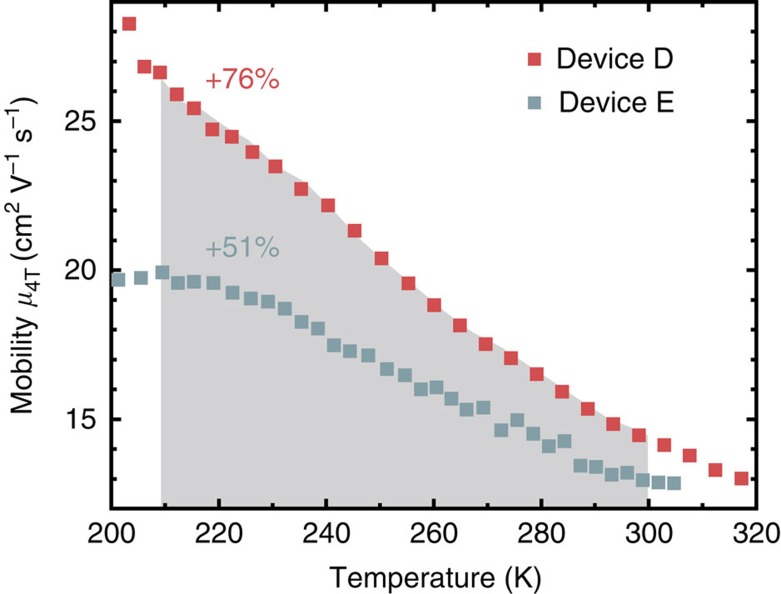
‘Band-like' transport in C_10_-DNBDT-NW single-crystal thin films. The increasing mobility at lower temperature shows the dominating influence of thermal fluctuations on charge transport. The increase by 76% within a decrease of temperature by 85 K (highlighted in grey) is in line with the calculations, which showed that the reduction of thermal fluctuations due to strain is equivalent to a temperature reduction by 85 K.

To prove that a reduction of temperature indeed increases the mobility, the temperature dependence of the four-terminal mobility (*μ*_4T_) in C_10_-DNBDT-NW was measured in two samples (Fig. 5). They show a mobility increase when cooled down, indicative of charge transport dominated by the thermal fluctuations of the individual molecules. As temperature decreases by 85 K, the mobility increases by 76% and 51% in devices D and E, respectively. We observed a sample-to-sample variation of the increase which presumably stems from the residual static disorder in the semiconducting thin-film crystal, leading to two competing mechanisms influencing the carrier mobility: band-like transport and multiple trap-and-release transport. Band-like transport is characterized by the negative temperature dependence of mobility due to the reduction of thermal fluctuations^36^,^37^,^38^,^39^,^40^. When static disorder is present, however, the ratio of the number of free carriers in relation to the total number of carriers decreases with decreasing temperature, which results in a positive temperature dependence of the mobility. Because of this effect, a band-like temperature dependence of the mobility can usually only be observed in a high-temperature regime turning into an activation-type mobility in a low-temperature regime^41^. Since device D has higher mobility and exhibits band-like transport over a wider temperature range than device E, device D obviously has less static disorder than device E. For this reason, we focus on device D, which exhibits more intrinsic characteristics of band-like transport. The increase in mobility by 76% when cooling the device by 85 K is quantitatively consistent with the large increase of mobility while bending the substrate. These results show that the two methods of reducing the amplitude of the molecular fluctuations, strain and cooling give quantitatively the same results, thus supporting the hypothesis that the fluctuations are indeed the origin of the increase in mobility. This finding shows that applying strain to organic semiconductors can enhance their mobility even beyond the mobility in a single crystal in equilibrium.

### Comparison between band and transfer integral calculations

So far, we analysed the effect of strain on charge transport by calculating the changes of the band structure under strain, which is a reasonable approach in the present system where the charge carriers are distributed over several molecules. A different approach is to analyse the transfer integrals between the molecules, which, strictly speaking, is more applicable to hopping systems and polaronic systems. The advantage of this approach is that the transfer integrals, their fluctuations and changes under strain can be quantitatively compared for each vibrational mode. A detailed discussion of the procedure is given in the supporting information.

The result of this theoretical analysis is that the mobility is expected to increase by 77% on compression, which is in good quantitative agreement with the measured increase of up to 70%. The increase originates equally from an increase of the transfer integral due to compression of the crystal lattice and a suppression of molecular fluctuations. Therefore, these results also support the hypothesis that the reduction of the molecular fluctuations is a necessary ingredient to understand the large mobility increase in strained organic single crystals.

To conclude, we measure a large mobility increase in single-crystal OFETs after applying a uniaxial compressive strain via bending the substrates. Effects of the contacts, as well as the gate insulator on this modulation could be excluded. The static changes of the crystal structure and therefore the band structure are proved to be too small to explain the large increase in conductivity and mobility. We demonstrate that the reduction of thermal fluctuations of each individual molecule is crucial to explain the large mobility increase. These findings may open up a way to exploit strain to produce organic semiconductors on flexible substrates with high charge mobility that can be used in ultrasensitive bending sensors for soft robotics applications.

## Methods

### Sample preparation

Bottom-gate-top-contact devices were prepared. Polyethylene naphthalate (PEN) (Teijin Teonex Q51) with a thickness of 188 μm was used as a flexible substrate. The PEN substrate was first heated up to 150 °C and kept at this temperature on a hot plate for 3 h, then gradually cooled down. Each substrate was cleaned in acetone/IPA in an ultrasonic bath for 10 min. Then, a gold layer (50 nm) was vacuum deposited through a shadow mask to form the gate electrode. As a gate dielectric, a fluorinated polymer (EPRIMA AL Asahi Glass Co.) was spin coated and cured at 150 °C for 30 min. The organic semiconductor C_10_-DNBDT-NW was synthesized in our laboratory. The single-crystal thin film was grown using the edge casting method. C_10_-DNBDT-NW was dissolved (0.025 wt%) in 3-chlorothiophene (Pi-Crystal Inc., Japan) at 120 °C. Then, the device was heated on a hot plate to 75 °C and a piece of glass was set on the device. A drop of the solution was sustained by the glass to control the evaporation, so that single crystals grew perpendicular to the glass. The crystals grow along the *c*-axis, which is also the channel direction. Afterwards the sample was annealed in vacuum at 100 °C for 10 h to get rid of residual solvents. This method produced large single crystals. We verified that the channel was single-crystalline using a polarized optical microscope. Then, a gold layer (50 nm) was vacuum deposited through a shadow mask to form the source and drain electrodes and probes for the four-terminal measurement. The organic semiconductor around the channel and the electrodes was laser-etched to suppress leakage current. The typical width (*w*) and length (*l*) of the channels were 50 × 150 μm, and the typical distance of the two probes for the four-terminal measurement from the source (or drain) electrode was 50 and 100 μm. The typical width and length of the substrates were 1.0 × 1.0 cm. The channel was placed at the centre of the device.

### Electrical and XRD measurements

The device was then set on a compression holder. Thin gold wires were attached with indium solder to metal sticks on the holder. The wires were attached onto the electrodes with carbon paste or gold paste and the metal sticks were connected to a semiconductor parameter analyser. With this process, the gold wires remained attached to the electrodes during bending of the substrates. The substrate was bent by compressing from the side. Bending radius and strain were calculated from the horizontal distance from one side of the bent substrate to the other with an accuracy of 0.1 mm. The electric characteristics were measured with a Keithley 4200-SCS semiconductor parameter analyser in dark and ambient condition. The mobility was calculated following the equations *μ*_2T, Lin_=(*l*/*w*)(1/*C*_i_*V*_D_) (∂*I*_D_/∂*V*_G_) (in linear regime), *μ*_2T, Sat_=(*l*/*w*)(2/*C*_i_)(∂|*I*_D_|^1/2^/∂*V*_G_)^2^ (in saturation regime) and *μ*_4T_=(1/*C*_i_)(∂*σ*/∂*V*_G_), where *C*_i_ is the capacitance of the gate dielectric and *σ* is the sheet conductivity measured with the four-terminal measurement. The capacitance of the gate dielectric layer was measured at a voltage bias of 0 V, and at a frequency of 1 kHz before applying the strain.

Temperature-dependent four-terminal mobility measurements were taken in a cryostat (Oxford Instruments) using an Agilent E5270B. The temperature was swept several times to ensure reproducibility of the mobility increase. Transmission XRD measurements were performed using a Rigaku 191R diffractometer system with a Cu target. The flexible samples have been strained using a self-built sample holder.

### Optimization of crystal structures and calculation of band structures

We used DFT taking vdW interaction into account. VASP was employed using the rev-vdW-DF2 functional^33^. Plane waves were used as the basis with a kinetic energy cutoff of 875.0 eV. The *k*-point sampling was done in 2 × 2 × 2 (Monkhorst–Pack mesh). The lattice constant along the *c*-axis in the unit cell was shortened according to the applied strain. The *b*-axis was elongated under strain, as has been found in the XRD measurement. The *a*-axis was kept constant. Then the arrangement of the atoms was optimized to find the energy minimum. As a result of the calculations, the optimized structure and the bandwidth of the HOMO band and the effective mass of the band under each strain were obtained.

### Calculation of molecular fluctuations

The molecular fluctuations in the unstrained/strained crystals were estimated using the Gaussian 09 software package^35^ employing DFT. The basis set was 6–31G(d) and the functional was PBEPBE corrected with Grimme's D2 (ref. 42) to include vdW interaction. The position and orientation of the core, without the alkyl chains, of a set of seven nearest-neighbour molecules (Fig. 4a) from the optimized crystal structure without and with strain were extracted and used as a starting point to calculate the influence of molecular fluctuations. The centre molecule was then shifted to represent the displacement due to the vibration of the molecule. Then the total energy of the system was recalculated. The molecule was translated along the c*- and *b*-axes, and rotated around the *a*-axis. The total energy of the system reflects the potential energy of the molecular fluctuations in the crystal.

## Additional information

**How to cite this article:** Kubo, T. *et al*. Suppressing molecular vibrations in organic semiconductors by inducing strain. *Nat. Commun.* 7:11156 doi: 10.1038/ncomms11156 (2016).

## Supplementary Material

Supplementary Information Supplementary Figures 1-10, Supplementary Tables 1-3, Supplementary Notes 1-2 and Supplementary References
