# Supplementary material for: Suppressing molecular vibrations in organic semiconductors by inducing strain
Source: Nat Commun. 2016 Apr 4;7:11156. doi: 10.1038/ncomms11156 (PMC4822010; doi:10.1038/ncomms11156)
Supplement: Supplementary Information — Supplementary Figures 1-10, Supplementary Tables 1-3, Supplementary Notes 1-2 and Supplementary References [file ncomms11156-s1.pdf]

Supplementary Information

Supplementary Figures

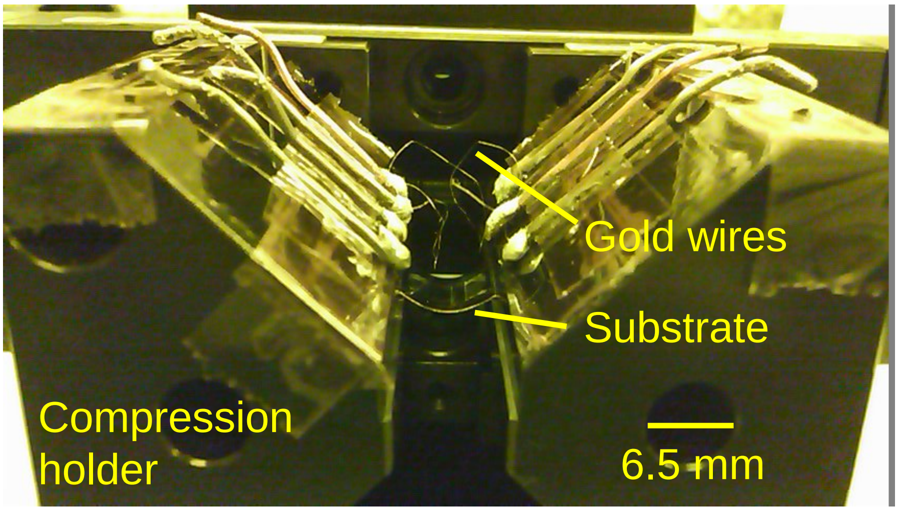

Supplementary Figure 1 | Picture of the compression holder.

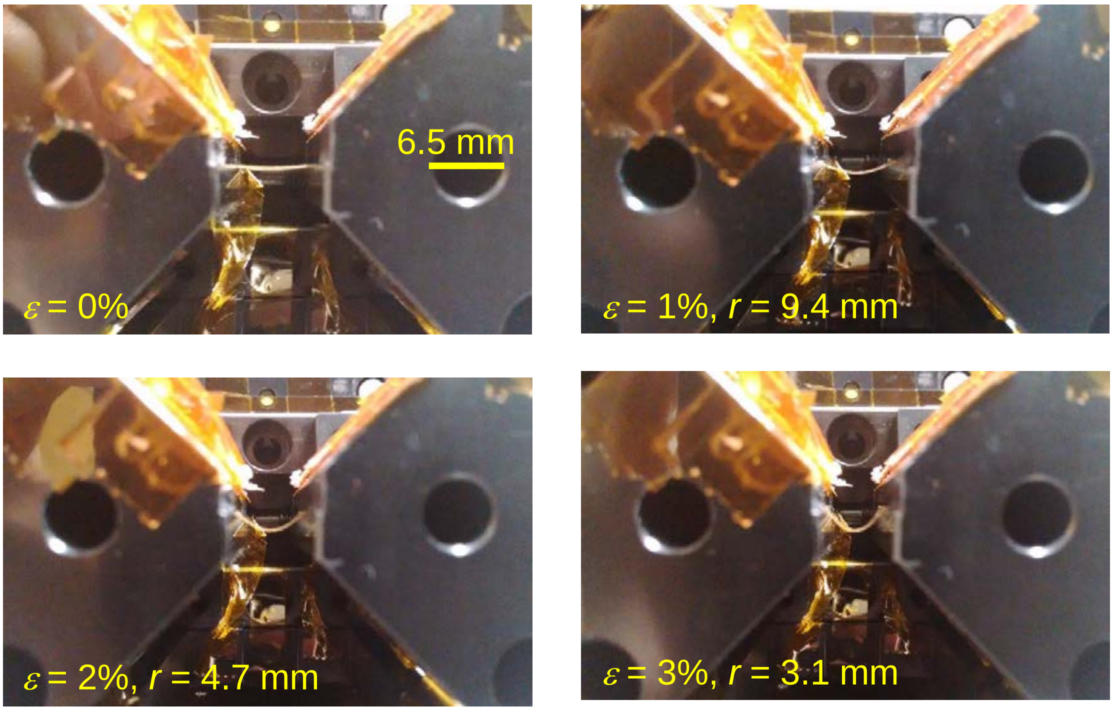

Supplementary Figure 2 | Pictures of the substrates bent by the compression holder for strain in the range from 0% to 3%. Strain and radius of the curvature calculated from equation (1) and (2) in the main text are shown.

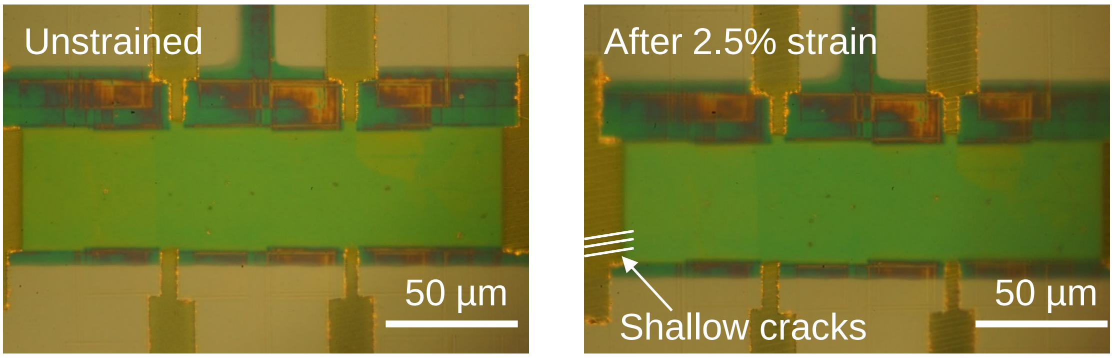

Supplementary Figure 3 | Optical microscope image of the channel before and after applying strain. The channel is a single crystal, i.e. no grain boundaries in the channel. Shallow cracks (< 10 nm deep), barely visible on the gold electrodes, exist along the channel (the *c* axis) before strain is applied. The cracks become more visible after applying strain, probably due to a slight expansion of the crystal perpendicular to the channel (the *b* axis) during bending (Poisson effect) as confirmed by the XRD measurements. These shallow cracks do not interfere with the conductivity of the channel. To conclude, when applying a strain below 2.5%, only small changes are visible in the semiconducting layer in the channel region, and therefore, strain up to 2.5% can be applied repeatedly.

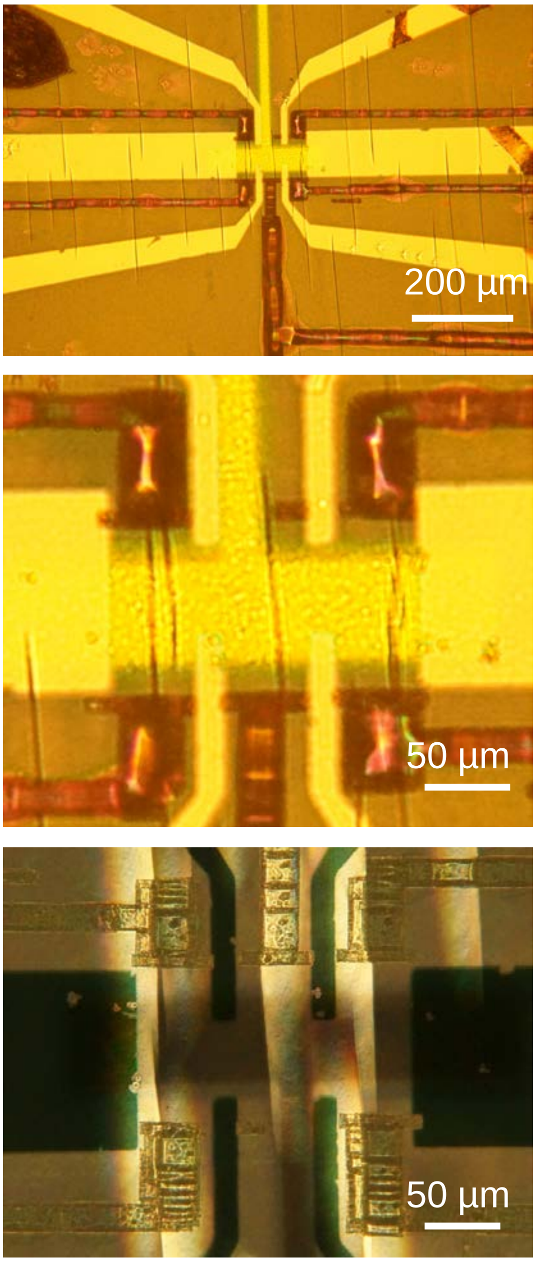

Supplementary Figure 4 | Deep cracks after applying extreme strain. Cracks perpendicular to the channel in a non-working device after applying more than 3% strain are shown here by top two reflected light microscopic images with different magnification and bottom one transmitted light microscopic image. At a strain beyond 2.5%, the devices start to fail due to cracks forming perpendicular to the channel. These cracks actually consist of fault pairs where the central part is lifted up by over 100 nm, similar to “horst fault blocks” in geology. The third image, where the uplifted parts are brighter, shows that the horst fault blocks extend over several tens of micrometers. The uplift by over 100 nm, as measured by AFM, shows that the whole dielectric layer cracked and probably delaminated from the substrate, destroying the channel.

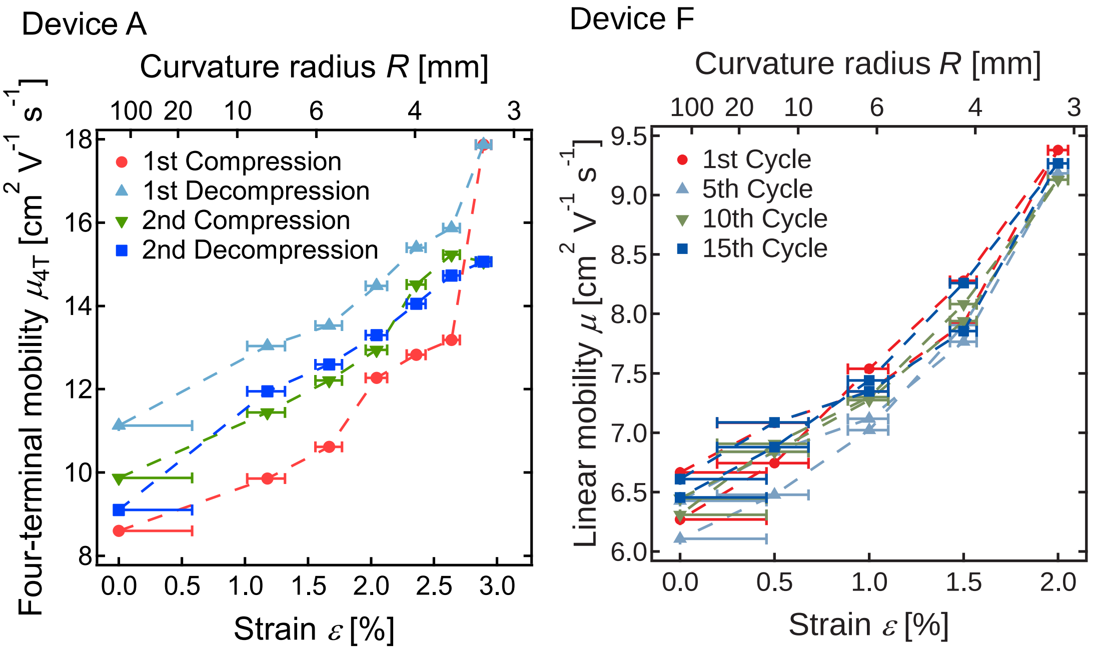

Supplementary Figure 5 | Reproducibility of the mobility under repeatedly applied strain. Reproducibility of the four-terminal mobility evolution under two compression cycles for device A (left figure). Horizontal error bars indicate the reading error of 0.1 mm of the compressed distance dL. The second compression cycle was conducted about half a day after the first compression cycle. The difference in mobility at the end of the first decompression and at the beginning of the second compression may derive from the recovery of the slight plastic deformation of the PEN substrate. Reproducibility of the mobility evolution under successive compression cycles for device F (right figure). Horizontal error bars indicate the reading error of 0.1 mm of the compressed distance dL. For device A, compression and decompression shows a substantial hysteresis while for device F, the hysteresis is much smaller. This results show that when applying a strain below 2% the effect of the strain is totally reversible and the mobility increase is reproducible over several cycles.

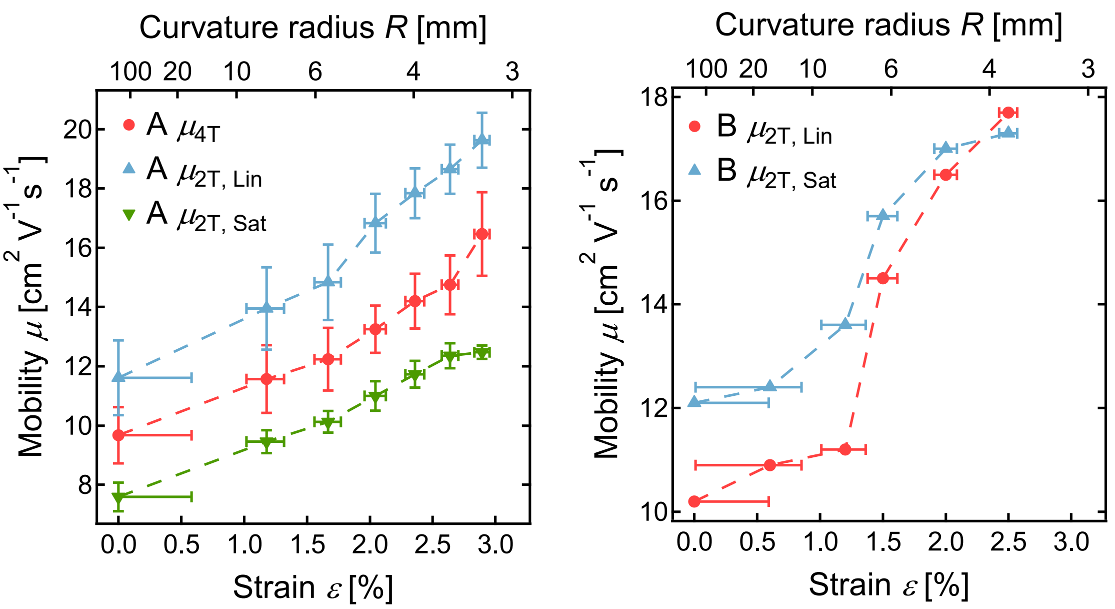

Supplementary Figure 6 | Linear, saturated and four-terminal mobility under strain for device A, and linear and saturation mobility under strain for device B. Horizontal error bars indicate the reading error of 0.1 mm of the compressed distance dL.

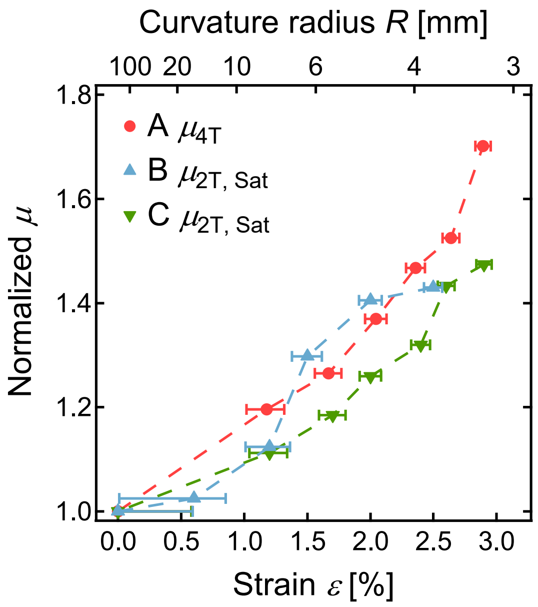

Supplementary Figure 7 | Comparison of the normalized mobility under compressive strain for the three devices. The relative change in mobility is similar, even though the initial mobility is different, indicative of a common origin of the mobility increase. Horizontal error bars indicate the reading error of 0.1 mm of the compressed distance dL.

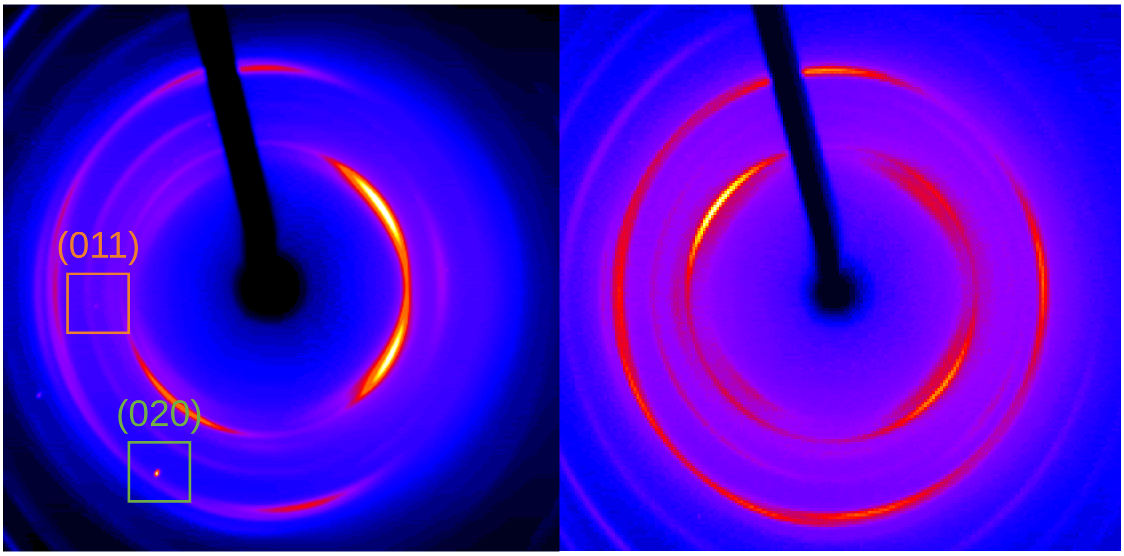

Supplementary Figure 8 | Transmission XRD patterns. Unstrained sample with the crystal plane peaks indicated (left). Unstrained sample with PEN substrate and polymer insulator only (right). The broad ring-like background patterns are therefore due to the scattering in the substrate and insulators, which are clearly distinct from the single-crystal peaks shown in Fig. 3 in the main article.

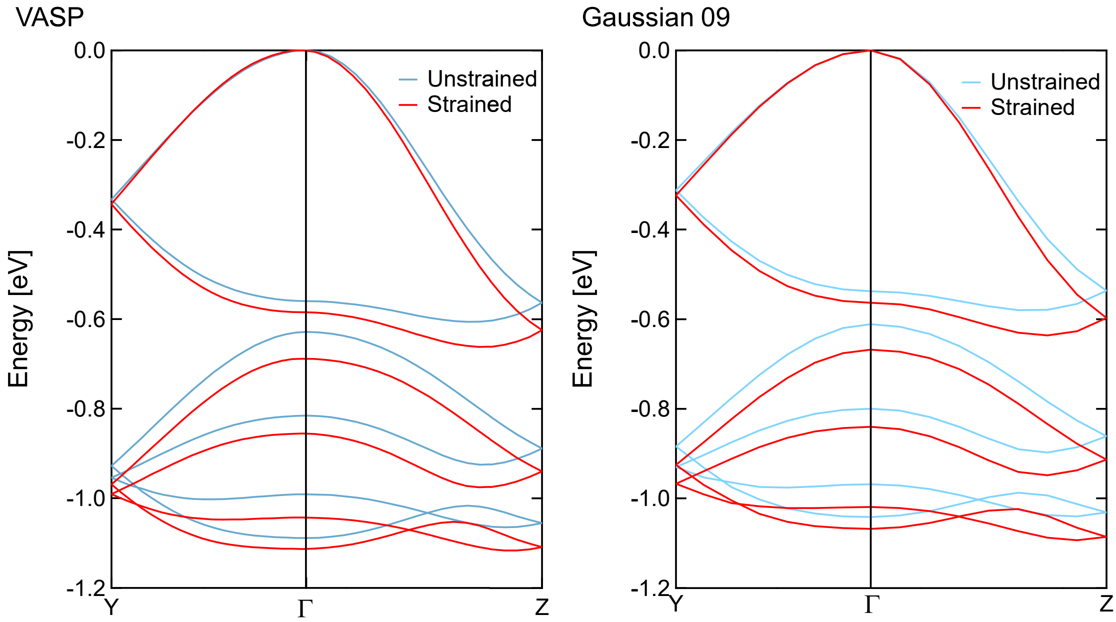

Supplementary Figure 9 | Band structure of unstrained and strained (strain = 3%) C_{10}-DNBDT-NW calculated with two different programs (VASP and Gaussian 09). The bandwidth and the effective mass of the holes are calculated from the band with the highest energy in the diagram (HOMO band), along the *c* axis (Γ–Z direction). The Γ–Y direction corresponds to

the *b* axis.

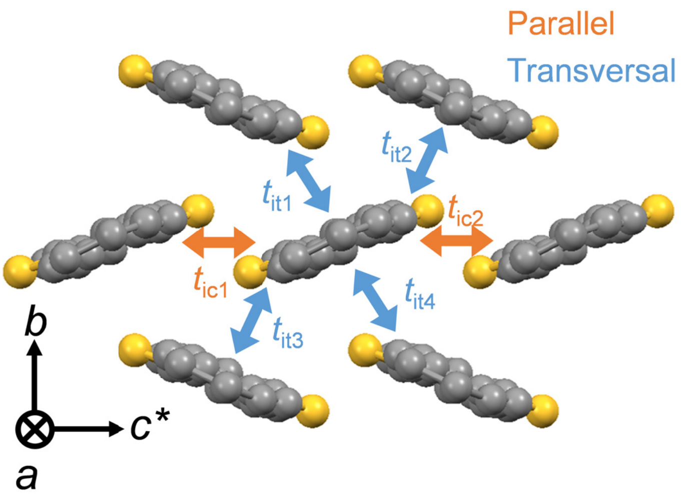

Supplementary Figure 10 | Calculations of the transfer integrals t_{ij}. *i* stands for three vibrational modes, *j* stands for pairs of neighboring molecules in the crystal.

Supplementary Tables

Supplementary Table 1 | The bandwidth and the effective mass of the carrier hole along the *c* axis as calculated by two different programs. m_0 is electron mass.

Bandwidth [eV]

Software	Strain = 0%	Strain = 3%	Change
VASP	0.562	0.624	11.0%
Gaussian 09	0.537	0.598	11.4%

Effective mass (m^*/m_0)

Software	Strain = 0%	Strain = 3%	Change
VASP	0.877	0.882	0.570%
Gaussian 09	0.867	0.873	0.692%

Supplementary Table 2 | The amplitude of three molecular vibration modes in unstrained and strained C_{10}-DNBDT-NW crystals.

Vibrational mode	Strain = 0%	Strain = 3%	Change [%]
Rotation around *a* [°]	2.01	1.86	-7.49
Translation along *b* [Å]	0.0607	0.0554	-8.71
Translation along *c* [Å]	0.0963	0.0809	-16.0

Supplementary Table 3 | The transfer integral *t* and its fluctuation Δ*t* in unstrained and strained C_{10}-DNBDT-NW crystals.

Parallel to the *c* axis

	Vibrational mode	Strain = 0%	Strain = 3%	Change
t [meV]		55.097	65.790	19.4%
Δ*t* [meV]	Rotation around *a*	0.879	0.351	-60.1%
	Translation along *b*	0.329	0.220	-33.0%
	Translation along *c*	4.96	4.38	-11.8%
	All modes	5.05	4.40	-13.0%
Δ*t*/*t*	All modes	9.24%	6.72%	-27.2%

Transversal to the *c* axis

	Vibrational mode	Strain = 0%	Strain = 3%	Change
t [meV]		76.767	82.260	7.16%
Δ*t* [meV]	Rotation around *a*	3.01	3.72	23.5%
	Translation along *b*	7.28	7.24	-0.461%
	Translation along *c*	7.04	5.55	-21.2%
	All modes	10.6	9.85	-6.71%
Δ*t*/*t*	All modes	14.0%	12.2%	-13.0%

Supplementary Notes

Supplementary Note 1 | Band structure calculations

The band structure of unstrained/strained C_{10}-DNBDT-NW was calculated using two different methods to ensure the accuracy of the results and hence the conclusions. As a first method, we used the VASP software with the rev-vdW-DF2 functional using plane waves as a basis set with a kinetic energy cutoff of 875.0 eV. The *k*-point sampling was done in 2 × 2 × 2 (Monkhorst-Pack mesh). As a second method we used the Gaussian 09 software with the PBEPBE functional and 6-31G(d) as a basis set. The crystal structure used to extract those parameters was optimized with VASP, as mentioned in the main text. The two calculation methods give slightly different results regarding the shape of the band diagram (Supplementary Fig. 9), and the absolute values for the bandwidth as well as the effective mass (Supplementary Table 1). The relative changes between the unstrained and strained crystal structure, however, are essentially the same. Therefore, the conclusion drawn from the band structure calculations does not depend on the calculation method. More specifically, the change in effective mass is the same for both models and can thus not explain the large mobility increase under strain.

Supplementary Note 2 | Calculations of transfer integrals and their fluctuation

In the main text we calculated the effective mass of the charge carrier using the band transport picture. Here we discuss the changes of the transfer integrals upon applying strain and their fluctuations due to molecular vibrations. It needs to be pointed out, that in a system with extended wave functions the effective mass is the relevant parameter describing the mobility, whereas the results discussed here from the transfer integrals are more applicable to a hopping system, which we do not have. Nevertheless, the discussion of transfer integrals brings additional insights into the mechanisms governing charge transport. As mentioned in the main text, the position and orientation of the core, without the alkyl chains, of a set of 7 nearest-neighbor molecules from the optimized crystal structure without and with strain were extracted (Supplementary Fig. 10). Here the transfer integrals *t* at the equilibrium state were calculated for the pair of transfer integrals parallel to the *c* axis and transversal to the *c* axis (i.e. along the *b* axis). Then, for the three vibrational modes mentioned in Supplementary Table 2 as well as in the main text, the center molecule was displaced by the amplitude of each vibrational mode i (≤ 3). The transfer integrals t_{ij} of the pair j (= t1, t2, t3, t4, c1, c2; $j \leq N = 6$) were calculated and the shift of the transfer integrals Δt_{ij} are averaged into the root mean square Δt_i as depicted in equation (1) to show the absolute value of the fluctuation of the transfer integral. The fluctuation of the transfer integrals are also normalized and summarized for all vibrational modes according to equation (2) and (3).The contributions to the fluctuation from each vibrational mode is summed up. Those values and the effect of strain on them are listed in

Supplementary Table 3.

$$\Delta t_{\mathrm{i}} = \sqrt{\frac{1}{N}\sum_{\mathrm{j}} \Delta t_{\mathrm{ij}}{}^{2}} \quad (1)$$

$$\Delta t = \sqrt{\sum_{\mathrm{i}} \Delta t_{\mathrm{i}}{}^{2}} \quad (2)$$

$$\Delta t/t = \sqrt{\sum_{\mathrm{i}} \left(\frac{\Delta t_{\mathrm{i}}}{t_{\mathrm{i}}}\right)^{2}} \quad (3)$$

The magnitude of the fluctuations of the transfer integral ($\Delta t/t$) is on the order of 10%. This is in line with recent findings by Fukami *et al.* for pentacene, where $\Delta t/t$ is between 11 and 18%[1]. These results can also be compared to the calculations by Troisi *et al*. They use a more involved approach which captures all possible degrees of freedom of the fluctuations and their correlation in detail. They found that for very small molecules the fluctuations are on the same order of magnitude as the transfer integral itself [2].The ratio is lower for larger and more complex molecules, but still larger than the results presented here[3,4]. Therefore, the simplifications used in our approach are expected to result in an underestimation of the fluctuations. The relative change of the fluctuations upon compression, however, can be estimated using our approach.

Upon compressive bending by a 3% strain, the transfer integral increases by 19% for the pair parallel to the *c* axis and by 7.2% for the pair transversal to the *c* axis (Supplementary Table 3). At the same time, the fluctuations reduce by 6.3 to 13% depending on the direction.

Finally, the tight binding approximation is used to calculate mobility from the transfer integrals. Therefore, the results are not directly comparable to the results from the band structure calculations. In the tight binding model, the mobility is proportional to $(a\ t/\Delta t)^2$ derived from Fermi's golden rule[5], where a stands for the crystal axis length. If the ratio of Δt to t becomes smaller, the scattering of the charge carriers is suppressed. As in Supplementary Table 3, the increase of $(a\ t/\Delta t)^2$ parallel to the channel is about 77%, where static changes of the crystal lattice and the molecular vibrations contribute equally to the mobility increase. This is in very good quantitative agreement with the experimental results where a mobility increase of up to 70% was observed. Therefore, in both models, in the band transport picture and in the transfer integral picture, the suppression of the molecular fluctuation is a crucial ingredient to explain the mobility increase under strain.

Supplementary References

1. Fukami, T. *et al.* Correlation between thermal fluctuation effects and phase coherence factor in carrier transport of single-crystal organic semiconductors. *Appl. Phys. Lett.* **106,** 143302 (2015).

2. Sleigh, J. P., McMahon, D. P. & Troisi, A. Effect of the intermolecular thermal motions on the tail of the electronic density of states in polyacene crystals. *Appl. Phys. A* **95,** 147–152 (2008).

3. Vehoff, T., Baumeier, B., Troisi, A. & Andrienko, D. Charge Transport in Organic Crystals: Role of Disorder and Topological Connectivity. *J. Am. Chem. Soc.* **132**, 11702–11708 (2010).

4. Troisi, A. & Orlandi, G. Dynamics of the intermolecular transfer integral in crystalline organic semiconductors. *J. Phys. Chem. A* **110,** 4065–4070 (2006).

5. Ishii, H., Kobayashi, N. & Hirose, K. Strong anisotropy of momentum-relaxation time induced by intermolecular vibrations of single-crystal organic semiconductors. *Phys. Rev. B* **88,** 205208 (2013).
